# The mismatch negativity as an index of cognitive decline for the early detection of Alzheimer’s disease

**DOI:** 10.1038/srep33167

**Published:** 2016-09-12

**Authors:** Manuela Ruzzoli, Cornelia Pirulli, Veronica Mazza, Carlo Miniussi, Debora Brignani

**Affiliations:** 1 Departament de Tecnologies de la Informació i les Comunicacions, Center for Brain and Cognition, Universitat Pompeu Fabra, Barcelona, Spain; 2 Cognitive Neuroscience Section, IRCCS Centro San Giovanni di Dio Fatebenefratelli, Brescia, Italy; 3 Center for Mind/Brain Sciences (CIMeC), University of Trento, Italy

## Abstract

Evidence suggests that Alzheimer’s disease (AD) is part of a continuum, characterized by long preclinical phases before the onset of clinical symptoms. In several cases, this continuum starts with a syndrome, defined as mild cognitive impairment (MCI), in which daily activities are preserved despite the presence of cognitive decline. The possibility of having a reliable and sensitive neurophysiological marker that can be used for early detection of AD is extremely valuable because of the incidence of this type of dementia. In this study, we aimed to investigate the reliability of auditory mismatch negativity (aMMN) as a marker of cognitive decline from normal ageing progressing from MCI to AD. We compared aMMN elicited in the frontal and temporal locations by duration deviant sounds in short (400 ms) and long (4000 ms) inter-trial intervals (ITI) in three groups. We found that at a short ITI, MCI showed only the temporal component of aMMN and AD the frontal component compared to healthy elderly who presented both. At a longer ITI, aMMN was elicited only in normal ageing subjects at the temporal locations. Our study provides empirical evidence for the possibility to adopt aMMN as an index for assessing cognitive decline in pathological ageing.

Auditory mismatch negativity (aMMN) is an event-related potential (ERP) component occurring approximately 100–200 ms after a detectable change (deviant stimulus) in a repetitive and predictive sequence of sounds (standard stimuli)^1^. aMMN appears maximal at the central-frontal electrodes with an inversion of polarity at the mastoids, which is consistent with neural generators located in the temporo-frontal network^2^,^3^. aMMN recorded from temporal electrodes is associated with the encoding of the physical features of the stimuli and the maintenance of the sensory memory trace; while aMMN recorded from frontal electrodes has been linked to involuntary capture of attention triggered by the occurrence of the deviant tone^4^,^5^,^6^,^7^. The aMMN elicitation arises from an automatic comparison between the deviant sensory input and the sensory-memory trace representing the preceding stimuli^8^. MMN is also considered an index of the efficiency of the auditory system to extract regularities in a sequence of sounds and to detect abnormalities based on predictions, according to the predictive coding theoretical framework^9^. aMMN is commonly used in clinical settings for indexing (i) auditory discrimination accuracy, (ii) sensory–memory duration^5^, and (iii) general cognitive decline^10^.

Recording aMMN at different inter-stimulus intervals (ISI) (typically, short ISI are less than 500 ms, whereas long ISI are more than 2 sec) is an experimental modulation used to investigate the accuracy of sensory memory encoding (short ISI) and the integrity of maintenance of sensory information (long ISI). By gradually extending ISI, the aMMN eventually vanishes, which enables one to assess sensory-memory duration. It has been found that in healthy participants, acoustic memory decays after a few seconds because aMMN is no longer elicited if the ISI is longer than 10 seconds^11^,^12^.

Because aMMN is elicited in the absence of direct control of voluntary attention, it is considered an automatic orienting towards salient events, and for this reason, it is particularly useful for the investigation of clinical populations in which prolonged sustained attention tasks are difficult to perform. Interestingly, Näätänen and colleagues have recently proposed MMN as an index of the cognitive decline occurring in a large number of different neurological and neuropsychiatric diseases^10^,^13^,^14^. Irrespective of their different aetiologies and symptomatologies, most of these disabilities share a functional deficiency of the auditory-frontal cortex network of auditory discrimination.

AD is a neurodegenerative and progressive dementia characterized by a gradual cognitive decline, principally affecting memory and other cognitive abilities, such as attention, language and executive functions^15^. In addition, with disease progression, behavioural symptoms such as delusions and agitation, and changes in personality or mood disturbances may also occur^16^. At the pathogenetic level, the core hallmarks of AD are the accumulation of beta-amyloid plaques and neurofibrillary tangles, which are associated with neuronal loss, synaptic loss and brain atrophy (for a review^17^,^18^). Therefore, AD dementia is considered part of a concoction of clinical and biological phenomena.

Many studies support the presence of a long preclinical phase of AD, estimated to begin decades before any clinical symptoms occur^19^,^20^,^21^. In fact, mild cognitive impairment (MCI) is a clinical syndrome characterized by objective cognitive decline with overall preserved everyday activities^22^. According to the symptomatology, two principal subtypes of MCI patients are identified: amnestic MCI (aMCI), in which memory appears to be prominently impaired^23^,^24^, and non-amnestic MCI, in which there are mild cognitive deficits in several domains without memory component (e.g., visuospatial skills, attention, language). aMCI has long been considered a prodromal phase of AD^23^,^25^, as clinical and longitudinal evidence indicates that a large portion of patients diagnosed with aMCI subsequently develop AD^26^,^27^. AD poses a tremendous socioeconomic burden on families and on national health systems worldwide. Several experiments are currently underway, investigating effective interventions in people with MCI that would enable practitioners to intervene early in the trajectory of the disease and thus slow down or even halt progression to AD. For this reason, it could be crucial to identify a reliable clinical marker for an early diagnosis of the disease (e.g. ref. 28). Modifications of aMMN latency or amplitude across MCI and AD patients may be an ideal marker to evaluate a functional impairment of the fronto-temporal network in the continuum of a disease affecting primarily memory.

The literature is quite consistent in suggesting that the mechanisms responsible for the automatic detection of deviant stimuli and for the memory-trace formation are preserved in AD patients in the auditory^29^,^30^,^31^ and visual domain (considering the visual counterpart to MMN (vMMN)^32^,^33^. The memory traces, however, decay faster in AD patients compared to age-matched healthy subjects, as revealed in a study by Pekkonen and colleagues^34^, in which a smaller aMMN amplitude was reported at 3 s of ISI. To our knowledge, no other studies have explored aMMN in AD patients at longer ISI as index of pathologically shortened sensory memory duration.

On the other hand, the existing evidence on aMCI patients is contradictory, principally due to the scarcity of studies and the more heterogeneous manifestations of the disease. To date, only two studies have evaluated aMMN in aMCI patients^35^,^36^. Mowszowski and colleagues^35^ presented binaural pure tones at a 500 ms stimulus onset asynchrony and found reduced aMMN amplitude over the temporal areas in the aMCI group compared to the healthy control group. These results were considered to reflect an impairment of the early stages of the information processing. Lindìn and colleagues^36^ used a more complex auditory-visual attention-distraction task, in which the interval between auditory stimuli was 2.35 s. They did not found any significant difference between aMCI patients and healthy control subjects when comparing the aMMN elicited by standard (i.e., tone bursts, 1000 Hz) and deviant (i.e., tone bursts, 2000 Hz) stimuli. However, they did observe significantly smaller aMMN amplitude in the aMCI than in the control subjects when comparing standard tones with novel stimuli (different each time: glass crashing, ringing, etc.). This effect was reported only for the middle-aged subgroup (between 50 and 64 years of age) while no difference in the aMMN amplitude was found for adults 65 years and older. This data, although hard to interpret, may suggest some sort of weakening of echoic memory trace in aMCI patients.

In our study, we evaluated, by means of aMMN, the potential alterations of auditory sensory memory in AD and aMCI patients compared to a control group of normal elderly subjects. We used a fast paradigm^37^,^38^ that reduces the recording time compared to the classic paradigm by about one-third, suited perfectly to our purpose of testing clinical populations. To identify possible differences between groups in encoding acoustic stimuli and/or in maintaining the representation of such stimuli over time, we compared the aMMN elicited at short vs. long inter-trial intervals (ITI, 400 vs. 4000 ms; it should be noted that in the present paradigm, the ITI definition overlaps with the classical ISI definition). Based on the previous evidence, we expected to observe aMMN in AD patients at short, but not at long ITI, as 4000 ms should exceed their sensory-memory duration. Crucially, aMCI patients, who are commonly considered to be midway between normal ageing and dementia, could already show an abnormal aMMN component similarly to AD patients. This would allow discriminating normal and pathological ageing in the early phases.

## Methods

### Participants

In total, 18 healthy elderly people, 12 aMCI patients and 19 AD patients took part in the experiment. Data from healthy participants of a previous experiment (see ref. 38) were used as control. Patients were recruited in the outpatient facility of the National Institute for the Research and Care of Alzheimer’s disease at the IRCCS Centro San Giovanni di Dio Fatebenefratelli (Brescia, Italy). The clinical diagnosis of aMCI or AD followed the standard clinical criteria^26^,^39^ and was based upon a complete neurological, neuropsychological and neuroimaging assessment.

Inclusion criteria was comprised of the following: (i) a Mini Mental State Examination score (MMSE) > 24 (corrected for age and level of education) for healthy elderly and aMCI patients^40^ and (ii) MMSE < 24 and a stable dose of a cholinesterase inhibitor for at least six months prior to the onset of the study for AD patients. Exclusion criteria, instead, included evidence of significant medical illness or substance abuse that could impair cognitive functioning and any other major systemic, psychiatric or neurological illnesses. Furthermore, a brief hearing test was performed prior to the EEG recording to exclude subjects who presented peripheral sensory deficit.

All participants were submitted to a neuropsychological evaluation to assess the global cognitive functioning (MMSE), language comprehension (Token Test), memory (Digit Span; Spatial Span, Rey-Osterrieth Complex Figure- Recall, Rey Auditory Verbal Learning Test -Immediate and Delayed), constructional and visuo-spatial abilities (Rey-Osterrieth Complex Figure - Copy), attention and executive functions (Trail-Making Test A and B) (see table 1). All tests were administered and scored by neuropsychological professionals according to standard procedures^41^. Healthy elderly people who presented one or more pathological test scores were excluded from the study.

EEG recordings heavily compromised by muscular artefacts and/or eye movements were excluded from the analysis. The final composition of the groups was therefore as follows: 12 healthy elderly (7 males, mean age 69 ± 5.51 years), 8 aMCI (4 males, mean age 68 ± 6.27 years) and 12 AD (6 males, mean age 72 ± 4.49 years). The groups were matched for age (*F* (2, 29) = 2.303, p = 0.1) and education (*F* (2, 29) = 0.774, p = 0.5).

Written informed consent was obtained from all participants or from their caregivers prior to the beginning of the experiment. All experimental protocols were performed in accordance with the Code of Ethics of the World Medical Association. The experiment was approved by the Ethics Committee for research in human subjects of the IRCCS San Giovanni di Dio Fatebenefratelli, Brescia.

### Stimuli and Procedure

**Figure 1:**
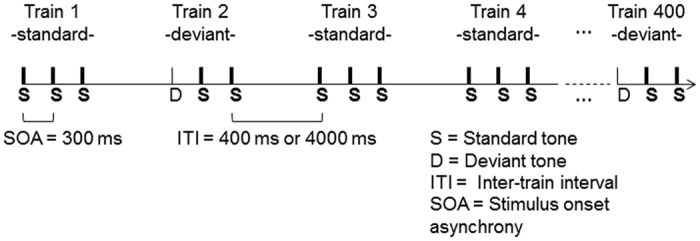
Schematic representation of auditory MMN paradigm. In total, 400 sequences (200 standard trains and 200 deviant trains) of 3 tones were presented randomly. The sequences differed only for the first tone, which could be standard (S = 75 ms, thick lines) or deviant (D = 25 ms, thin lines). The SOA between tones within the same train was 300 ms. The ITI between the beginning of the last tone of the previous train and the beginning of the next tone could be 400 (short) or 4000 ms (long) depending on the condition.

The stimuli and procedures were the same as those used in Grau *et al*.^37^ and replicated in Ruzzoli *et al*.^38^ (see Fig. 1). Sequences of three tones (train of tones) were presented binaurally with earphones. The sequences differed only for the first tone, which could be standard (50%) or deviant (50%). All the remaining tones were standard. The standard tone was a pure sine-wave tone of 700 Hz, with an intensity of 85 dB SPL (Sound Pressure Level) and a duration of 75 ms. The deviant tone had the same frequency and intensity as the standard tone but a different duration (i.e., 25 ms). The interval within the train of stimuli was 300 ms.

Each participant completed two separate experimental sessions in which different short (400 ms) or long (4000 ms) ITI were used. The order of presentation of the sessions was balanced across participants. In each session, 200 standard and 200 deviant trains of stimuli were randomly delivered. The 400-ITI and 4000-ITI sessions had a length of 7 and 32 minutes, respectively, including short pauses.

After positioning the EEG cap and earphones, participants sat in a comfortable chair in a dimly illuminated room. During the EEG recording, each subject was instructed to watch a silent film without subtitles, to ignore the auditory stimuli and to avoid extra eye movements and blinking.

### EEG recording and data processing

EEG signal was recorded from 19 electrodes set in an elastic cap (Electro-Cap International, Inc.), positioned according to the 10–20 International system (AEEGS, 1991). The electrode locations were Fp1, Fp2, F7, F3, Fz, F4, F8, T7, C3, Cz, C4, T8, P7, P3, Pz, P4, P8, O1, and O2. EEG signal was also recorded from left and right mastoids (M1, M2). Fpz was used as ground. The reference electrode was placed at the tip of the nose. The electro-oculogram (EOG) was recorded from two bipolar channels placed at the external side of both eyes for the horizontal EOG and above and below the right eye for the vertical EOG. Data were collected with a high cut-off filter of 80 Hz and digitalized at a sampling rate of 250 Hz (BrainAmp MRplus, BrainProducts GmbH, Munich, Germany). The impedance was kept below 5 kΩ.

Event-related potentials were obtained offline. The EEG recordings were filtered with a pass-band filter of 0.1–30 Hz and then divided into epochs, synchronized with the first tone of each sequence (standard or deviant). Each epoch was 500 ms in length, including 100 ms pre-stimulus baseline. Trials with eye movements, blinks and muscle artefacts were excluded from analysis, as well as trials in which the voltage exceeded ±75 μV at Fp1/2, Fz, F3/4, F7/8, Cz, C3/4, T7/8 and M1/2 locations. Average responses to deviant and standard tones were computed separately for each subject and for each ITI (400, 4000 ms). The responses to the last two tones, of each sequence of stimuli (both standard tones), were not analysed.

### aMMN Analysis

The MMN component was quantified by measuring mean amplitude of responses evoked by standard and deviant tones in the 150–180 ms time window. This temporal interval was estimated by visual inspection of the temporal window where aMMN occurred in the grand-average waveforms of each group and ITI condition, separately. Four electrode locations were considered for the analyses, which were considered as frontal (F3, F4) and temporal (M1, M2) aMMN components. Importantly, we expected an enhanced negativity to the deviant tone relative to the standard tone over the frontal site and the inversion of polarity over the temporal sites, as previously reported in the literature (Giard *et al*. 1995; Sams *et al*.^11^).

Mean amplitudes were analysed with a repetitive measures ANOVA with Group (Elderly, aMCI and AD) as between-subjects factor and Tone (standard, deviant), ITI (400 ms, 4000 ms), Area (frontal, temporal) and Hemisphere (left, right) as within-subjects factors. Considering the inversion of polarity between frontal and temporal areas, the presence of the aMMN component in the analysis should be reflected by the interaction between Tone and Area factors, or in their interaction with other factors. The Greenhouse-Geisser method was applied when appropriated.

### Correlation between aMMN and cognitive functioning (neuropsychological variables)

In order to highlight possible relations between aMMN and neuropsychological performance, we performed Pearson correlation coefficient analyses considering the amplitude of aMMN at 400 ITI (separately for frontal and temporal areas) and the neuropsychological test scores (MMSE, Token Test, Digit Span; Spatial Span, Rey Auditory Verbal Learning Test Immediate, Rey-Osterrieth Complex Figure- Recall, Rey-Osterrieth Complex Figure-Copy, Trail-Making Test A and B) in all the participants. All contrasts were two tailed and employed an alpha level of 0.05.

## Results

### aMMN

**Figure 2:**
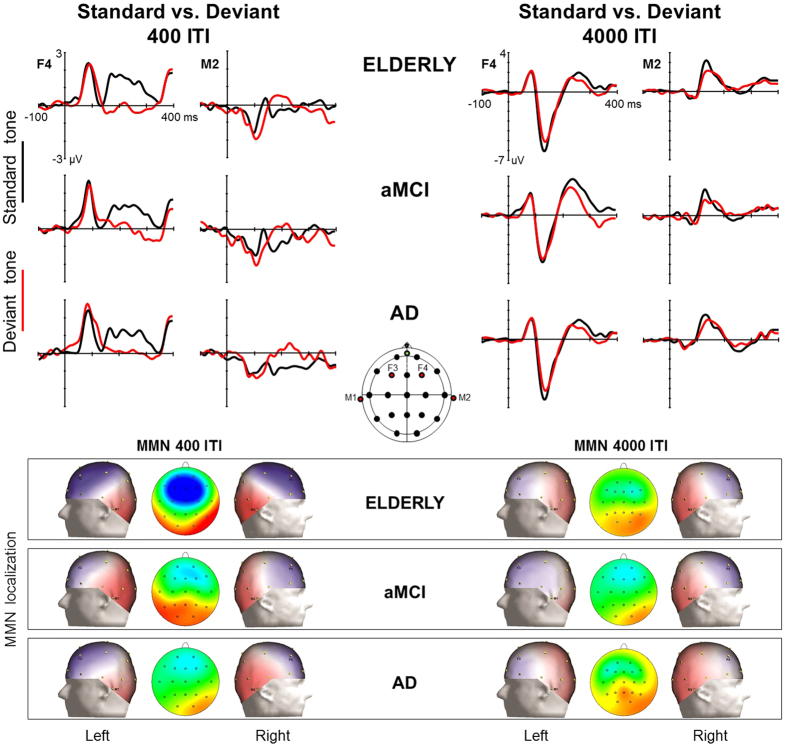
ERP to the standard and deviant tones and topographical maps of aMMN. At the top: grand-averaged waveforms elicited by standard (black) and deviant (red) tones in healthy elderly participants, aMCI and AD patients. On the left: 400 ITI condition; on the right: 4000 ITI condition. The grand-average response from the two electrodes F4 and M2 of the right hemisphere is reported for illustrative purposes. At the bottom: scalp topographies of the aMMN amplitude in the 150–180 ms time window for the three groups. The maps show the aMMN localized on the right and left side of the scalp for both ITI conditions. In the middle, analysed electrodes are represented in red.

Figure 2 shows the ERP to the standard and deviant tones and the corresponding topographical maps of the difference waves (i.e., aMMN) for healthy elderly, aMCI and AD group, separately. aMMN was evoked in frontal areas and in temporal regions (reversed polarity) as showed by the significant interactions Tone x Area (*F* (1, 29) = 36.15, p < 0.001). The interaction ITI x Tone x Area (*F* (1, 29) = 36.14, p < 0.001) suggested that the presence of aMMN was affected by the ITI factor. To better understand this effect, we performed repetitive measures ANOVAs with Group (elderly, aMCI and AD), Tone (standard, deviant), Area (frontal, temporal) and Hemisphere (left, right) separately for each ITI condition. The ANOVA on 400-ITI condition showed a highly significant Tone x Area interaction (*F* (1, 29) = 59.57, p < 0.001). Subsequent post-hoc analysis, corrected with Tukey’s HSD test, revealed a significant difference between standard and deviant tones (i.e., aMMN) in both frontal (p < 0.001) and temporal location (p < 0.001). Also the ANOVA on 4000-ITI condition indicated a significant Tone x Area interaction (*F* (1, 29) = 10.12, p = 0.003), but subsequent post-hoc analysis proved the presence of the aMMN in temporal (p = 0.013), but not in frontal (p = 0.48) areas.

The interaction ITI x Tone x Area x Group, which approached significance (*F* (1, 29) = 2.66, p = 0.08), indicated that this pattern could be different across the groups. With the aim of understanding in which areas and ISIs the aMMN emerged as difference between standard and deviant tones in every group, we performed subsequent repetitive measures ANOVAs separately for each group, with Tone (standard, deviant), ITI (400 ms, 4000 ms), Area (frontal, temporal) and Hemisphere (left, right) as factors. The ANOVA on healthy elderly showed significant interaction Tone x Area (*F* (1, 11) = 25.06, p < 0.001) and ITI x Tone x Area (*F* (1, 11) = 39.59, p < 0.001), revealing the presence of aMMN over both frontal (p < 0.001) and temporal (p < 0.001) areas in the 400-ITI condition, and a temporal (p = 0.02), but not frontal (p = 0.21), aMMN in the 4000-ITI condition. The ANOVA on MCI patients disclosed the same significant interactions as in healthy elderly [Tone x Area (*F* (1, 7) = 6.18, p = 0.04) and ITI x Tone x Area (*F* (1, 7) = 5.72, p = 0.048)], but post-hoc comparisons revealed the presence of the aMMN only in temporal areas (p = 0.020) in the 400-ITI condition (p > 0.1 in all the other comparisons). Similar results were found in the ANOVA on AD patients [Tone x Area (*F* (1, 11) = 10.30, p = 0.008) and ITI x Tone x Area (*F* (1, 11) = 5.78, p = 0.035)], who however showed a significant difference between standard and deviant tones only in frontal areas (p = 0.001) during the 400-ITI condition (p > 0.1 in all the other comparisons). To evaluate possible differences in the mean amplitude of aMMN across the groups, we compared directly the difference waves between standard and deviant tones (i.e., aMMN) only in the areas and ITI where the aMMN was actually present (i.e., healthy elderly vs. AD patients in the frontal areas at 400-ITI; healthy elderly vs. MCI patients in the temporal areas at 400-ITI). No significant group effects concerning aMMN amplitude emerged (all p’s > 0.7).

Finally, the main ANOVA showed the significant interaction Tone x Area x Hemisphere (*F* (1, 29) = 8.40, p = 0.007), which also interacted with Group (*F* (1, 29) = 3.33, p = 0.049). Subsequent post-hoc analysis, corrected with Tukey’s HSD test, revealed that, independently by the ITI, healthy elderly and AD groups showed a frontal and temporal aMMN both in the left and in the right hemispheres, while the aMCI group showed aMMN only in the right hemisphere (frontal and temporal areas).

### Neuropsychological Test

**Figure 3:**
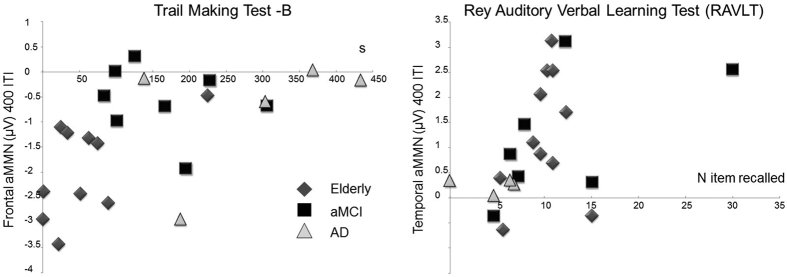
Correlation between aMMN amplitude and neuropsychological tests. (**a**) Scatterplot correlation between frontal aMMN amplitude (y-axis, in μV) and the Trail Making Test-B score (x-axis, in seconds). (**b**) Scatterplot correlation between temporal aMMN amplitude (y-axis, in μV) and the Rey Auditory Verbal Learning Test (RAVLT) score (x-axis, number of items recalled).

Neuropsychological test scores were corrected for age and education. The groups significantly differed in the scoring of MMSE (F(2, 29) = 25.44, p < 0.001), Spatial Span (*F* (2, 27) = 5.51, p < 0.01), Token test (*F* (2, 28) = 24.86, p < 0.001), Rey-Osterrieth Complex Figure- Copy (*F* (2, 27) = 17.8, p < 0.001) and Recall (*F* (2, 26) = 18.73, p < 0.001), and Trial-Making Test A (*F* (2, 23) = 8.25, p < 0.005) and B (*F* (2, 20) = 12.58, p < 0.001). Post hoc comparisons are highlighted in Table 1. Please note that the scoring of some tests is missing for some patients who were unable to complete the entire neuropsychological evaluation. Figure 3 shows correlations between aMMN amplitude and neuropsychological test scores (where present) for healthy elderly, aMCI and AD participants at 400-ITI condition only. We did not perform the correlation analysis for the 4000-ITI condition because aMMN was present only in healthy elderly in temporal location. As reported in Table 2, the results showed a significant correlation between frontal aMMN and Trail Making Test B (n = 23, r = 0.531 p = 0.01) and between temporal aMMN and Rey Auditory Verbal Learning Test Delayed (n = 22, r = 0.465 p = 0.03). For completeness, we also reported statistical tendencies in the correlation between frontal aMMN and Token Test (n = 31, r = −0.319 p = 0.08) and between temporal aMMN and Digit Span scores (n = 31, r = 0.336 p = 0.07).

## Discussion

In this study, we analysed the frontal and temporal aMMN components elicited by duration deviant sounds in two different inter-train intervals (ITI: 400 vs. 4000 ms). We compared data from three groups of participants: healthy elderly, aMCI and AD patients. The primary aim was to provide empirical evidence for testing the strategic possibility to adopt aMMN as a parametric index able to characterize the progression of memory impairment from normal ageing to AD.

Our results showed interesting dissociations between the three populations of participants. In fact, at the short ITI (400 ms), AD patients showed a frontal but not temporal aMMN, whereas aMCI patients showed a temporal, but not a frontal, aMMN, in comparison to healthy elderly, who showed aMMN in both the frontal and temporal areas. At 4000 ms ITI, aMMN was recorded only in the control group at the temporal area. When aMMN was present in two groups in the same conditions (healthy elderly and AD patients: frontal areas at 400-ITI; healthy elderly and MCI patients: temporal areas at 400-ITI) it showed the same amplitude across the groups.

Our results partially contradict previous evidence showing that early stages of sensory memory were not affected by dementia when short intervals between stimuli (from 500 to 2000 ms) are adopted^29^,^30^,^31^,^32^,^33^,^42^. It should be noted that most of the studies on aMMN focused on pitch change discrimination, while the present one investigated aMMN elicited by duration deviant sounds. The literature on aMMN in normal ageing suggests separate cortical generators for aMMN to pitch and duration deviants^4^,^5^. Several studies consistently found a preserved aMMN to pitch changes but a reduced aMMN to duration deviants^7^,^43^,^44^,^45^ in healthy older individuals. The only two studies, which investigated aMMN to duration deviance in AD^43^,^46^ and in non-specified dementia^47^, actually found an unaffected aMMN compared with normal ageing. However, more investigations are needed to definitively exclude that aMMN elicited by pitch and duration changes are differently compromised in dementia patients.

In our study, we dissociate between the frontal and temporal components of aMMN. Even if there is not a clear relationship between an electrical field observed on the scalp and the brain regions giving rise to that field^48^, consistent evidence suggested different neural generators for the two inverted-polarity aMMN subcomponents. Specifically, aMMN recorded at temporal electrodes arises from neural sources localized in the supratemporal cortex, while MMN obtained at frontal electrodes reflects activity from the inferior frontal cortex^5^,^8^,^49^,^50^,^51^,^52^,^53^,^54^,^55^. The temporal component of aMMN is associated with sensory memory encoding (short ITI) and the maintenance of sensory information (long ITI)^8^. The frontal component has been linked to attentional aspects of change detection^5^,^51^ and more specifically, to the efficiency of the auditory system to extract regularities and to detect abnormalities based on predictions^9^. Garrido *et al*.^9^ proposed the predictive coding framework to interpret MMN that combines the change-detection^4^,^5^, the adaptation hypothesis^7^ and the model-adjustment hypothesis^56^ in a probabilistic perspective of perceptual optimization. The authors suggested that auditory perception is based on hierarchically and reciprocally organized neural systems, where the abstractions from higher cortical areas (i.e., frontal) should be matched with the data from the lower cortical areas (i.e., temporal) through backward connections. At the same time, through forward connections and prediction error signals, lower cortical structures try to adjust the predictions from higher cortical areas based on the actual data. This mechanism lasts until the prediction error signal is negligible and the abstractions from higher cortical areas are optimized^9^,^57^. Most of the studies reporting that automatic discrimination of auditory changes is not affected in AD patients evaluated only the frontal aMMN^29^,^30^,^34^. In line with these results, in the present study, AD patients showed that the frontal aMMN was comparable to healthy elderly, suggesting a preserved predictive coding system. aMMN, however, was absent over the temporal areas in the AD group.

AD has been defined as a disconnection syndrome^58^,^59^. Indeed, amyloid accumulation is followed by alterations in resting state functional connectivity at first and then by cortical atrophy^60^. Several large-scale neural networks have been explored in AD, providing evidence for a widespread reduction of both intra- and inter-network connectivity, which worsened as AD severity increased^61^. The default mode network (DMN), the most active network in absence of task demand, has been extensively studied in AD^27^, showing that several regions of the DMN are among the earliest to show abnormal amyloid deposition^62^,^63^,^64^. The DMN includes frontoparietal midline structures, parts of lateral temporal and parietal cortices, and the medial temporal cortex^65^,^66^. The evidence reported above suggests that the supratemporal cortex, which is the area where the neural generator of the temporal component of aMMN has been localized, is part of the DMN and shows an abnormal functional connectivity with other brain areas in AD patients. Some studies, for instance, have found a decreased connectivity in AD patients relative to controls between the superior temporal area (BA 22) and the hippocampus^67^,^68^. The passive listing paradigm adopted in our paradigm might have been activated the DMN, although participants were not in the classic resting-state condition. Consequently, the absence of the aMMN over the temporal areas observed in the present study in AD group can be ascribed to the pathological functional connectivity occurring in the declared phase of AD. The inferior frontal cortex that generates the frontal component of the aMMN is not part of the DMN and consistently, in our data, the frontal aMMN is preserved in AD patients. This result, however, is surprising because the frontal aMMN should also be reduced as result of a decreased functional connectivity between frontal and temporal areas and because substantial evidence of amyloid plaques accumulation has been reported in the frontal areas too^69^,^70^,^71^. Interestingly, Opitz and colleagues^49^ proposed a specific role of the frontal MMN generator in tuning the sensory system according to processing demands. They suggested that the activation of a frontal contrast enhancement system increases with decreasing distinctiveness of sound input. Accordingly, the presence of the frontal aMMN in AD patients might be ascribed to the low functioning of the temporal aMMN generator, which has difficulty in discriminating stimuli. Increased recruitment of the prefrontal regions in AD patients has been observed during memory task performance and has been suggested as a not task specific compensatory mechanism that reflects a general adaptation to loss of cognitive resources^61^,^72^.

Overall, what we observed at short ITI in AD patients is that the higher frontal areas are still able to elaborate predictions, but the temporal areas fail to manage low level processing. Exactly the opposite effect occurred in aMCI patients at short ITI. The sensory memory encoding (temporal aMMN) was preserved, while the frontal component was compromised. The pathophysiological alterations of AD begin early in the progression of the disease, even in the preclinical phase, before any cognitive symptom appears^60^. Similar to AD, aMCI patients also show patterns of decreased functional connectivity between regions of the DMN^73^,^74^,^75^. We argue that, given the earlier stage of impairment, the supratemporal cortex might still be preserved in aMCI compared to AD patients, such that they showed a temporal aMMN component comparable to that recorded in the healthy elderly. Indeed, an hyperactivation over the temporo-parietal and medial temporal regions has been reported in MCI patients compared to controls during memory tasks^59^,^76^,^77^,^78^. This pattern has been explained as a compensatory mechanism acting as cognition begins to be impaired in early AD pathology and diminished with further disease progression^79^. According to the contrast enhancement view^49^, activation of the frontal generator of aMMN critically depends on the interaction between superior temporal cortex and inferior frontal cortex. In conditions of normal acoustic stimulation, we expect healthy subjects to show a balanced activation of the frontal and temporal generators of aMMN. In AD patients, the low functioning of the temporal generator could be compensated by a hyper-activation of the frontal source. Conversely, in aMCI patients the pattern might reverse by an over-activation of the temporal generator, which might explain the absence of the frontal aMMN. Interpreting changes of the neural dynamics over the course of AD pathology however, is only speculative. Evidence suggests that the progression of the disease is associated with a nonlinear trajectory of activity alterations: decreases in activation in some areas are accompanied by evidence of increased neural activity in other regions, hinting at a more complex underlying pattern of network level dysregulation rather than simply at a universal reduction in network activity^61^. Due to the lack of investigations on aMMN in aMCI patients, a comparison of our findings with the literature is difficult. To date, there is only one study by Mowszowski and colleagues^35^ that examined aMMN generated by duration deviants at short ITI in aMCI population. They found that the aMMN response in aMCI was significantly attenuated in the temporal areas compared to healthy elderly, whereas there was no significant difference between groups in aMMN amplitude at frontal and central location. The inconsistency of findings may be due to the aetiological heterogeneity of aMCI patients. Current evidence, indeed, indicates that aMCI can result from AD pathology as well as from other non-AD aetiologies^80^. Further investigation on aMMN should characterize aMCI patients based on their biological profile in order to control for the heterogeneity of the sample.

The most relevant contribution of the present study concerns results of aMMN at the long ITI (4000 ms). In a previous study^38^ on changes in auditory sensory memory during physiological ageing, we found that the memory trace for the standard stimuli decayed more rapidly in healthy elderly respect to young and middle-aged individuals. At the long ITI, the elderly showed no frontal aMMN and a reduced temporal aMMN. Because the presence of the aMMN component is proved by the significant difference between the waves elicited by the standard and deviant tones, the temporal aMMN recorded in the healthy elderly reached no significant difference when compared with the larger aMMN generated in young and middle-aged participants^38^, but it did reach significance when compared with the small, if any, aMMN observed in the patients groups. In fact, in the present study, AD patients showed no reliable aMMN in any locations when the ITI was 4000 ms, in agreement with the previous literature. Pekkonen and colleagues^34^ found that aMMN in AD group was still present but reduced when using an interval of 3000 ms. It is plausible that such temporal interval corresponds to the boundaries of the sensory–memory duration in AD patients. Accordingly, the stimulus trace could still be present at 3000 ms, although already compromised, and it could be definitively decayed at longer temporal intervals. Pekkonen and colleagues^34^ however, investigated only the frontal aMMN; we do not know whether the aMMN was detectable at 3000 ms over the temporal regions. Consistent with our results concerning the short ITI, we can hypothesize that the aMMN is unlikely generated in AD patients over the temporal areas, at least for ITI superior to 400 ms.

Importantly, aMCI patients showed the same aMMN pattern observed in AD patients at long ITI. This means that, independent of the different dynamics affecting the encoding phase in the course of the disease, the impairment of echoic memory trace begins early in the progression of AD pathology. Therefore, our data support that aMMN investigated over both frontal and temporal areas at long ITI might be a sensitive index useful for an early diagnosis of cognitive impairment likely due to AD. This aspect is particularly valuable if we consider that the aMMN recording is non-invasive, low-cost and easy to conduct also for non-specialized staff members.

Our results are specific for the aMMN elicited by duration deviants, which has proven to be more sensitive than other types of deviants used in other clinical studies^81^. However, our findings have to be corroborated in studies with larger sample sizes. In addition, in order to identify a sensitive paradigm that might be adopted in the clinical routine, it is timely that further studies will explore both frontal and temporal aMMN at different ITIs ranging between 2000 and 4000 ms. Systematic investigation could reveal the most appropriate parameters, able to identify the earliest abnormalities (e.g., preclinical AD) and possibly to discriminate between different clinical populations (e.g., AD versus frontotemporal dementia). Moreover, repetitive sessions of aMMN could be a useful tool to monitor the progression of the disease and to predict the conversion of aMCI patients to AD. A recent study on comatose patients has reported that the deterioration in aMMN between two separate recording sessions was the most predictive index of clinical outcome^81^.

Finally, in line with our main purpose to test the reliability of aMMN as a neurophysiological index for memory loss progression from healthy elderly to AD, it is also important to underline that aMMN recorded at the short ITI correlated with some of the neuropsychological tests’ scores (see Fig. 3). In particular, we found that frontal aMMN significantly correlated with Trial Making Test-B. TMT-B^81^,^83^ is a neuropsychological test used for measuring visual attention and task switching (i.e., executive functions), which are considered frontal functions. Taking into account that TMT-B is measured in seconds and frontal aMMN has a negative value, this positive correlation showed that the more negative aMMN was, the faster the participants were to complete the test. In addition, in our sample, the Rey Auditory Verbal Learning Test (RAVLT) Recall correlated with the temporal component of the aMMN. Recall RAVLT is a test used to assess short-term auditory-verbal memory, rate of earning, learning strategies, retroactive, and proactive interference, presence of confabulation, of confusion in memory processes, retention of information, and retrieval. Because the score at TMT-B reflects the number of words recalled and temporal aMMN has a positive value, this positive correlation showed that the more positive the aMMN was, the better the performance at RAVLT. This evidence, although correlative, support the importance of the frontal and temporal aMMN as sensitive indices for evaluating frontal executive functions and memory integrity, respectively, in normal and pathological ageing.

In conclusion, our data supports the proposal by Nätäänen and colleagues^14^,^84^ that aMMN is a valuable index for assessing general cognitive decline in different neurological and psychiatric pathologies. Our data provide empirical evidence for the possibility to adopt aMMN in clinical settings as an index for memory decay in aMCI and AD patients and more generally in pathological ageing.

## Additional Information

**How to cite this article**: Ruzzoli, M. *et al*. The mismatch negativity as an index of cognitive decline for the early detection of Alzheimer’s disease. *Sci. Rep.* **6**, 33167; doi: 10.1038/srep33167 (2016).

## Figures and Tables

**Table 1:** Mean scores ( ± standard deviation) for demographic and neuropsychological variables for the three groups of participants (healthy elderly, aMCI and AD) and corresponding p-values of between-groups post-hoc comparisons (ns = non-significant).

	Elderly	aMCI	AD	cut-off	Elderly vs. aMCI	Elderly vs. AD	aMCI vs. AD
Gender, m/f	7/5	4/4	6/6	—	ns	ns	ns
Age, years	68.8 ± 5.5	68.1 ± 6.3	72.7 ± 4.5	—	ns	ns	ns
Education, years	9.5 ± 4.3	7.5 ± 2.7	7.7 ± 4.9	—	ns	ns	ns
**Screening for Dementia**							
MMSE	27.0 ± 1.8	26.4 ± 1.4	18 ± 4.9	24	ns	p < 0.01	p < 0.01
**Language Comprehension**							
Token Test	33.7 ± 2.0	31.28 ± 1.4	25.8 ± 3.8	26.5	ns	p < 0.01	p < 0.01
**Memory**							
Digit Span	7.5 ± 5.8	5.6 ± 0.9	4.5 ± 1.1	3.75	ns	ns	ns
Spatial Span	5.5 ± 0.8	4.3 ± 0.4	4.1 ± 1.5	3.55	p < 0.05	p < 0.01	ns
Rey Recall	18.0 ± 5.7	7.0 ± 6.0	4.2 ± 4.8	9.46	p < 0.01	p < 0.01	ns
Rey Auditory Verbal Learning Imm. test	45.2 ± 11.7	36.5 ± 17.5	28.0 ± 4.8	28.53	ns	ns	ns
Rey Auditory Verbal Learning Del. test	9.9 ± 2.8	11.9 ± 8.8	4.5 ± 3.1	4.69	ns	ns	ns
**Constructional and visuo-spatial abilities**							
Rey Copy	34.3 ± 1.8	25.1 ± 4.9	15.1 ± 11.5	28.87	p < 0.05	p < 0.01	p < 0.05
**Attention and Executive functions**							
Trail-Making Test A	24.4 ± 15.4	33.0 ± 10.4	92.4 ± 67.0	93	ns	p < 0.05	p < 0.01
Trail-Making Test B	58.4 ± 65.7	162.8 ± 76.7	285.8 ± 122.8	282	p < 0.05	p < 0.01	p < 0.05

**Table 2:** Pearson’s correlation coefficients (r) and associated p-values between aMMN amplitude (frontal and temporal) and neuropsychological test scores for healthy elderly, aMCI and AD participants at 400-ITI condition.

	MMN - ITI 400			
	Frontal MMN		Temporal MMN	
	*r*	*p-value*	*r*	*p-value*
MMSE	−0.047	0.79	0.136	0.46
Token Test	−0.319	0.08	0.188	0.31
Digit Span	−0.206	0.27	0.336	0.07
Spatial Span	−0.194	0.30	0.080	0.67
Rey Recall	−0.115	0.55	0.077	0.69
Rey Auditory Verbal Learning Imm.Test	0.087	0.70	0.126	0.58
Rey Auditory Verbal Learning Diff. Test	−0.137	0.54	0.465	0.03
Rey Copy	−0.082	0.66	0.179	0.34
Trial-Making Test A	−0.013	0.95	−0.094	0.65
Trial-Making Test B	0.531	0.01	−0.273	0.21

Significant values are in bold.
